# 3D Microscale Heat Transfer Model of the Thermal Properties of Wood-Metal Functional Composites Based on the Microstructure

**DOI:** 10.3390/ma12172709

**Published:** 2019-08-23

**Authors:** Yuan Chai, Shanqing Liang, Yongdong Zhou, Lanying Lin, Feng Fu

**Affiliations:** Research Institute of Wood Industry, Chinese Academy of Forestry, Beijing 100091, China

**Keywords:** microscale heat transfer model, microscopic thermal properties, wood-metal functional composite, finite element method

## Abstract

This study presents a model for simulating the microscopic heat transfer processes in a wood-metal composite material. The model was developed by analyzing the microstructure of experimental samples comprising a melted alloy impregnated in a wood matrix. According to the thermal parameters of the materials and the boundary conditions, an analytical model of microscale heat transfer was established using Abaqus finite element analysis software. The model was validated experimentally by comparing temperature curves obtained via simulation and experiments; the resulting correlation coefficient was 0.96557. We then analyzed the temperature distribution of the composite material with different cell geometries and heat transfer conditions (heat transfer direction and applied temperature). The thermal properties of the unit cell models were in good agreement with the general trends predicted by several heat transfer equations. This study provides a method for analyzing the microscale heat transfer process in wood-based composites. In addition, the model framework characteristics can be used to evaluate the heat transfer mechanism of impregnated modified wood.

## 1. Introduction

Wood-based composite materials are a new type of composite material that have unique advantages over monolithic materials, including such features as high strength, high stiffness, long fatigue life, low density, and adaptability to the intended function of the structure [1]. These composites can also overcome the limitations of a single material; specifically, wood has poor thermal conductivity (0.15 W·m^−1^·K^−1^) [2], whereas metals have high thermal conductivities. Therefore, using metals as thermally conductive fillers can considerably boost the thermal conductivity of wood in a wood-metal functional composite. As a result, wood-metal composites have important applications for geothermal flooring.

Wood–metal functional composites combine recyclable wood with one or more reinforcing alloys. The melted metal alloy acts as a transmission path within the wood, thereby overcoming the natural limitations of wood, such as poor dimensional stability, flammability, and discoloration [3,4], while exploiting the excellent mechanical and electrical properties of the metal, which include a high compressive strength, modulus of elasticity, hardness, wear resistance, fatigue resistance, and thermal conductivity [5,6]. Park et al. [7] employed a high-temperature and high-pressure process to impregnate wood with low-melting-point alloys (tin, bismuth, indium, and gallium) exhibiting high thermal conductivities. The prepared functional composites were predicted to have good dimensional stability, high flexural strength, and high thermal conductivity. Wan et al. [8] systematically studied the thermal conductivity properties of wood-metal functional composites. A low melting alloy sequence was fixed and separated in the wood tracheid, and the direction of electron transport was also fixed; therefore, the composites showed good anisotropy in terms of thermal, electrical, and mechanical properties. The thermal anisotropy ratio (κ_||_/κ_⊥_) for the composite was approximately 18, which indicates that such a composite material can be applied to multiple fields. However, no previous studies have evaluated the microscale heat transfer of wood-metal functional composites. Thermal transfer in the wood is complicated by its complex 3D microstructure [9,10], which also complicates the mode of heat transfer of the metal alloy [11]. Hence, computer simulations are required to model the microscale heat transfer within wood-metal composites.

Microscale studies on the thermal properties of wood are relatively rare. The thermal conductivity and steady state moisture diffusion coefficient of single wood cell layers have been studied in relation to their chemical components by Eitelberger et al. [12,13]. In addition, microscale thermal conductivity and moisture diffusion have been analyzed by evaluating microscale material parameters using analytical multiscale models. Lignocellulose plays an important role in the thermal conductivity of wood cell walls; i.e., the thermal conductivity of cell-scale lignocellulose is 0.73 W·m^−1^·K^−1^ at 317 K, whereas that of hardwood and softwood is 0.16 W·m^−1^·K^−1^ and 0.12 W·m^−1^·K^−1^, respectively [14]. Chen et al. [15] used transient thermal technology to characterize the thermal transport of lignocellulose at the cell scale from room temperature to 20 K, and discussed the relationship between thermal properties and temperature. Thoemen et al. [16] generated a digital model of wood microstructure from CT (Computed Tomography) images and used representative volume units to simulate the heat and mass transfer performance. Zhang et al. [17] applied material microstructure calculations, the finite element model (FEM), and homogenization theory to construct a wood microstructure model. They established the relationship between the macro- and micro-field and simulated the basic thermal properties of wood cells. Moreover, Xu et al. [18] studied the microstructural thermal conductivity of wood using scanning thermal microscopy.

However, the majority of microscale wood research has focused on its mechanical properties. For example, Koponen et al. [19,20] introduced a two-dimensional regular hexagonal honeycomb structure model and calculated the properties of the cell walls in early wood and late wood areas by assuming different densities. Kahle et al. [21] divided wood growth rings into three layers with fixed densities (early wood, transition wood, and late wood) and constructed an irregular hexagonal cell structure model. Qing et al. [22,23] established a hierarchical hexagonal model of wood microstructure and studied the effects of microfibril angle, cell wall thickness, cross-sectional cell shape, and wood density on the elastic properties of softwood. Fortino et al. [24] used the finite element method to quantitatively analyze deformation of the wood cells and cell walls and found a good agreement between deformation curves obtained via simulations and experiments. They also developed a model to simulate the effects of different charring temperatures on the material microstructure of wood [25]. Muzamal et al. [26,27] developed a comprehensive finite element model (FEM) including the unit cell, early and late wood, pits, and wood rays for evaluating the changes in spruce microstructural units during steam explosion treatments. Wood-only products are used in multiple fields; thus, some previous studies have conducted heat transfer modeling in order to understand the advantages and disadvantages of wood and its derivatives via numerical and experimental analyses [28,29]. However, modeling the microscopic heat transfer processes in a wood-metal composite material has not yet been accomplished.

The objective of this study was therefore to develop a method to calculate the temperature distributions in wood-metal functional composites from their microstructures. A microscale heat transfer model is developed that can extract the temperature and heat flux of the composite at any point in the model and at any time. The proposed approach is not limited to heat conduction in wood-metal composites; it can also be applied to different reinforcement phases in wood-matrix composites. The advantage of using a microscale heat transfer analytical model to simulate the properties of composite materials is that no experiments are required for sample preparation and detection. Thus, this approach is a fast and inexpensive way to correlate structural parameters and thermal properties.

## 2. Materials and Parameters

### 2.1. Preparation of Wood-Metal Functional Composites

**Figure 1:**
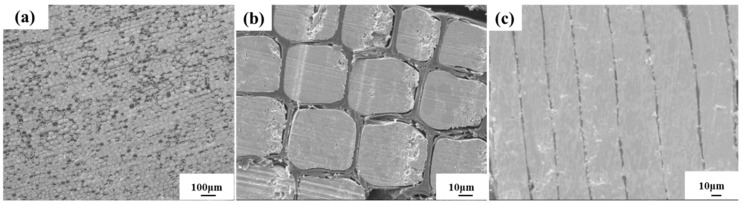
SEM images of wood-metal functional composites: (**a**,**b**) cross-section of sample and (**c**) tangential section of sample.

Wood–metal functional composites were prepared by impregnating Pinus radiata wood (density of 440 kg·m^−3^) with a low-melting-point alloy at high temperature and pressure (413.15 K and 1 MPa, respectively). The Pinus radiata was treated by a high-strength microwave before impregnation, where most of the tracheal channels were opened after treatment. The components of the alloy were Sn and Bi, with a melting point of 411.15 K and a density of 7100 kg·m^−3^. A complete sheet was cut along the cross-section of the composite by a blade, attached to a sample stage, and sprayed with gold. A cold-field emission scanning electron microscope (Hitachi S4800, 10 kV, Tokyo, Japan) was used to characterize the microstructure to define the geometry of the composite cells for the simulation. Representative SEM images are shown in Figure 1.

### 2.2. Thermal Parameters

The thermal parameters of the material are among the most important for the model, as well as the density, thermal conductivity, and specific heat of the material. Because the organic composition of all wood cell walls is the same, the cell wall density of each tree species is very similar. Wang [30] measured the density closest to the wood cell wall, which was approximately 1460 kg·m^−3^. For the thermal conductivity, λ_||_ and $\lambda_{⊥}$ denote the values parallel and perpendicular to the fiber direction. Softwood has the highest cellulose content (40%–45%), relatively high lignin content (26%–34%), and relatively low pentosan (7%–14%) content [31]. Thus, we assume that λ_||_ = 1 W·m^−1^·K^−1^ and $\lambda_{⊥}$ = 0.26 W·m^−1^·K^−1^ represents the thermal conductivity of cellulose 12. As research into the thermal properties of the cell wall is relatively rare, the specific heat of the cell wall is unknown. Thus, the formula obtained by Aseeva et al. [32] for the specific heat of oven-dried wood has been used for the calculation:

$$c(t)=1571(\frac{t}{293})$$

where *c* is the specific heat (J·Kg^−1^·K^−1^) and *t* is temperature (K).

The moisture content of oven-dried wood is theoretically equal to 0 and the cell wall density of wood is approximately 1460 kg·m^−3^. If there are no lumens or other voids in the wood, the oven-dried density is 1460 kg·m^−3^, which means that the mass of oven-dried wood is similar to the mass of the cell wall; thus, the specific heat of oven-dried wood can be approximated as the specific heat of the cell wall. According to the above equation, the specific heat of the cell wall and the oven-dried wood is approximately 1600 J·Kg^−1^·K^−1^ at room temperature (298.15 K). The thermal parameters of the alloy were measured by LFA 467 HyperFlash (NETZSCH, Selb, Germany) and DSC 214 (NETZSCH, Selb, Germany). Table 1 shows the thermal parameters of the wood cell wall and alloy.

**Figure 2:**
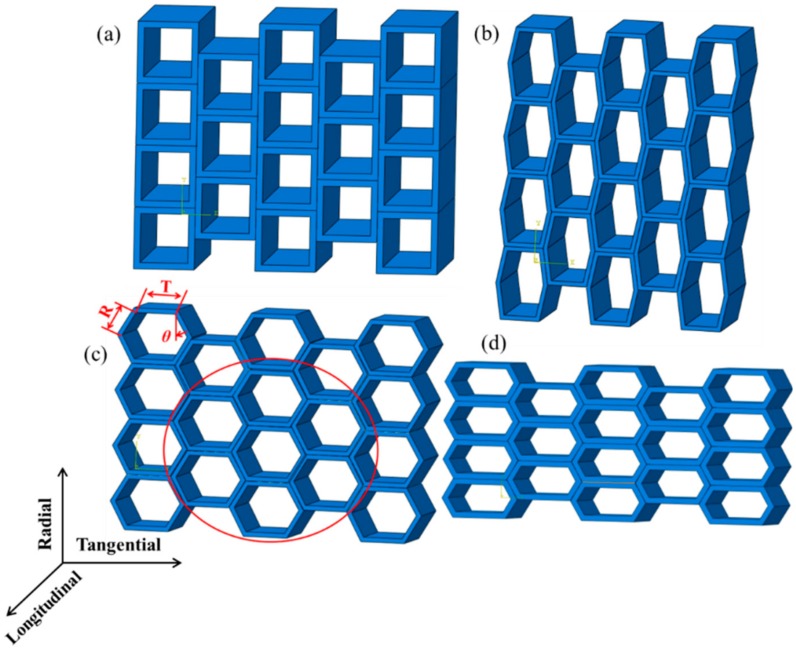
Schematic model of the wood cell. (**a**) *θ* = 0° and T/R = 1, (**b**) *θ* = 20° and T/R = 1, (**c**) T/R = 1.5 and *θ* = 30°, and (**d**) T/R = 3 and *θ* = 30°.

The heat transfer simulation was performed using cells with different sizes and geometries, as shown in Figure 2, where the length parameters T and R and the angle *θ* are defined. In the calculations, T/R values of 1, 1.5, 2, 2.5, and 3, and *θ* values of 0°, 10°, 20°, 30° and 40° were used to model the different cell morphologies of early wood and late wood. Figure 2 shows the wood cell models with *θ* = 0°, 20° (T/R = 1), and T/R = 1.5, 3 (*θ* = 30°). Referring to the studies of Qing [22] and Fortino [24], seven cells indicated by the red circle were selected for analysis. T was fixed at 30 μm according to the measurement results of several tracheids in the experimental SEM images. The thickness of the cell wall layers was 3.27 μm based on the results of a previous study [33].

## 3. 3D Microthermal Model

In this simulation, the finite element analysis software Abaqus 6.14-5 was used to simulate the temperature field distribution. The boundary conditions of the model were included as inputs for the calculations.

### 3.1. Solution of Boundary Conditions

According to the basic heat transfer equation, the boundary condition of the model was deduced. The law of conservation of energy was included in the model as the first law of thermodynamics. Specific to the heat transfer phenomenon, the sum of the heat entering the system through the boundary surface (*Q_1_*) and the heat generated in the system (*Q_2_*) is equal to the increase of the material energy *Q_3_* per unit time. Because there is no heat generated in the model of the wood-metal composite, *Q_2_ =* 0. Thus,

(1) $$Q_{1}=Q_{3}$$

#### 3.1.1. Heat Q_1_ Entering the System

**Figure 3:**
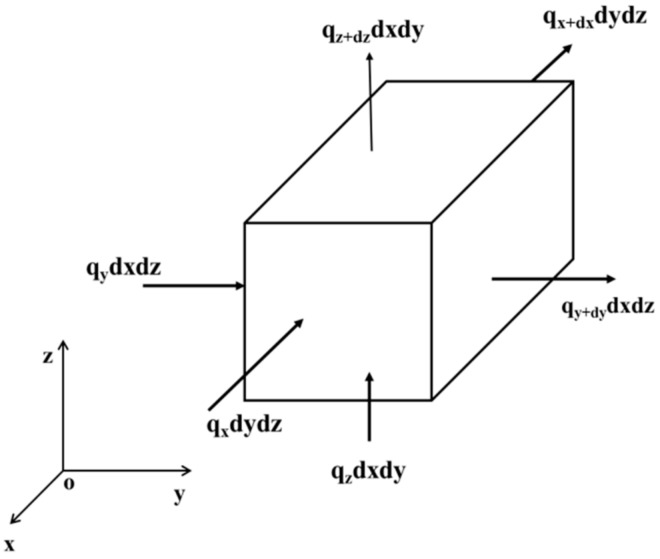
Energy conservation of a unit tracheid.

A tracheid in the wood-metal composite material was considered as a thermodynamic system d*V*, as shown in Figure 3. The heat flow from the left side to the tracheid along the y direction per unit time is $q_{y}dxdz$, whereas that from the right side is $q_{y+dy}dxdz$. Thus, the heat retained in the system per unit time along the *y* direction is as follows:

(2) $$Q_{1y}=(q_{y}-q_{y+dy})dxdz$$

If the temperature field *t* and heat flux *q_y_* are continuous in a compact solid, the equation can be written in the form of an expanded Taylor series at *y*, when the system d*V* tends to zero:

(3) $$q_{y+dy}=q_{y}+\frac{\partial q_{y}}{\partial y}dy$$

According to Fourier’s law:

(4) $$q_{y}=-\lambda \frac{\partial t}{\partial y}$$

Then,

(5) $$Q_{1y}=\lambda \frac{\partial^{2}t}{\partial y^{2}}dydxdz$$

Similarly, the heat entering the system in *x* and *z* directions per unit time can be written as follows:

(6) $$Q_{1x}=\lambda \frac{\partial^{2}t}{\partial x^{2}}dxdydz$$

(7) $$Q_{1z}=\lambda \frac{\partial^{2}t}{\partial z^{2}}dzdxdy$$

Then,

(8) $$Q_{1}=Q_{1x}+Q_{1y}+Q_{1z}=\lambda \frac{\partial^{2}t}{\partial x^{2}}dxdydz+\lambda \frac{\partial^{2}t}{\partial y^{2}}dydxdz+\lambda \frac{\partial^{2}t}{\partial z^{2}}dzdxdy$$

#### 3.1.2. Increase in Material Energy Q_3_

*Q_3_* represents the increase in thermal energy per unit time in the d*V* system:

(9) $$Q_{3}=\rho c\frac{\partial t}{\partial \tau }dxdydz$$

By substituting *Q_3_* and *Q_1_* into Equation (1), we obtain the following:

(10) $$\lambda (\frac{\partial^{2}t}{\partial x^{2}}+\frac{\partial^{2}t}{\partial y^{2}}+\frac{\partial^{2}t}{\partial z^{2}})=\rho c\frac{\partial t}{\partial \tau }$$

where *λ* is the thermal conductivity (W·m^−1^·K^−1^); *ρ* is the density (kg·m^−3^); *c* is the specific heat (J·Kg^−1^·K^−1^); *t* is the temperature (K); and *τ* is time (s). In this model, the external hot air mainly transfers heat to the material through thermal radiation, whereas heat transfer inside the material occurs via heat conduction. Only one side was heated in each simulation, so the heat transfer process was approximated as one-dimensional heat conduction along a certain direction (in this case, the *x* direction), and there was no internal heat source inside the material. Hence, the heat conduction equation was simplified as follows:

(11) $$\lambda \frac{\partial^{2}t}{\partial x^{2}}=\rho c\frac{\partial t}{\partial \tau }$$

These equations include initial conditions and boundary conditions. The material was at ambient temperature, *t*_0_, before the test. Assuming that the temperature distribution of the entire material is uniform and equal *t*_0_, the initial conditions are as follows:

(12) $$t(x){|}_{\tau =0}=t_{0}$$

Heat transfer between the material surface and the hot air occurs via thermal radiation under the heating conditions. When the heating temperature is fixed, the boundary conditions can be expressed as:

(13) $$-\lambda \frac{\partial t}{\partial x}=\varepsilon \sigma (t^{4}-t_{f}^{4})$$

where *ε* is the integrated emissivity coefficient; *σ* is the Stefan–Boltzmann constant with a value of 5.67 × 10^−8^ W·m^−2^·K^−4^, and *t_f_* is the heating temperature (K).

### 3.2. Model Development

The model was simulated using a transient non-linear thermal analysis method, where the finite element type was a three dimensional 8-node brick element (C3D8), divided as a whole into two areas and then each had its own property attached. Proper arrangement of the mesh seeds was ensured to allow meshing of the element [34]. The material was approximated by one-dimensional heat conduction by heating on one side and neglecting convective heat transfer on the heated surface. Therefore, the heated surface of the material was defined by thermal radiation with a radiance of 0.8, whereas the other surfaces were considered non-heated and defined by convection with a convective heat transfer coefficient of 5 W·m^−2^·K^−1^. Finally, a Stefan–Boltzmann constant of 5.67 × 10^−8^ W·m^−2^·K^−4^ and absolute zero temperature (0 K) were selected in the “edit attributes” option of the modeling software. The thermal conductivity of the material was defined by its theoretical properties. In the modeling process, parameters such as the total loading time and boundary conditions must be set. In the step module, a transient analysis was selected and a reasonable heating time of 120 s was set, which was attained via constant trial. In addition, the initial temperature was first applied to the material. The ambient temperature during the test was applied to each node of the material through the predefined field and the temperature was loaded according to a predefined temperature. 

During the process of establishing the model, it was assumed that: 1) the wood cells had no pits; 2) the thickness of the cell walls in early and late wood were the same (as the thermal conductivity of the wood cell wall is much lower than that of the alloy); and that 3) there was no tylosis in the cell cavity.

**Figure 4:**
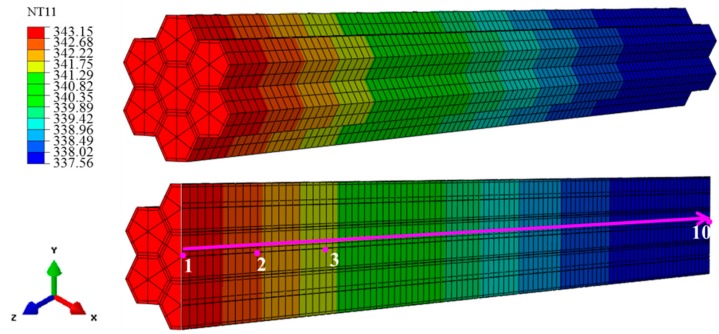
Diagram defining the temperature distribution of the model at 120 s, with temperature measurement points indicated by numerals. Red color indicates the location closest to the heating surface and blue color indicates the location farthest from the heating surface.

**Figure 5:**
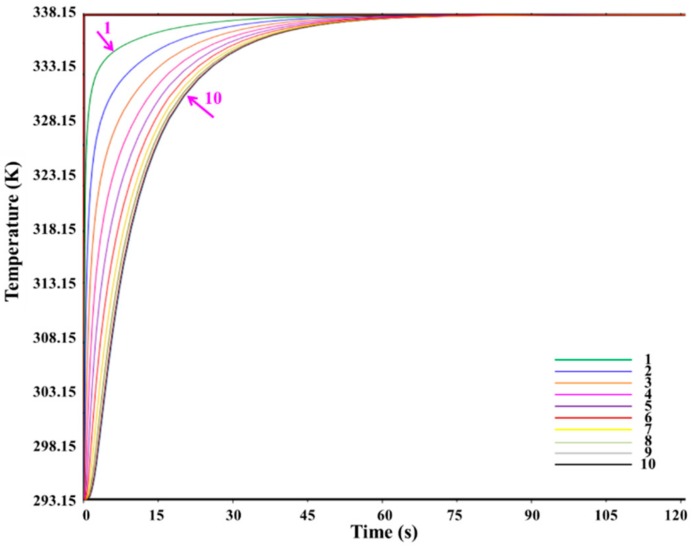
Change of temperature with time at 10 points along the arrow indicated in Figure 4, where 1 is closest to the heating surface and 10 is farthest from the heating surface.

**Figure 6:**
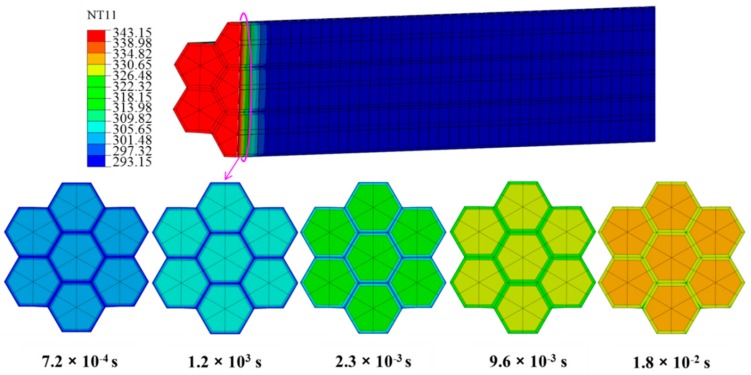
Temperature distributions at the beginning of heat transfer.

Figure 4 shows the temperature distribution for heat transfer at a time of 120 s, where the lower image shows the longitudinal cross-section. Figure 5 shows the temperature as a function of time at 10 points along the arrow indicated in Figure 4, where 1 is closest to the heating surface and 10 is farthest from the heating surface. Figure 6 shows the temperature distribution at the beginning of heat transfer. Here, T and R were 30 μm, *θ* was fixed at 30°, and the applied temperature was 343.15 K. Note that the coordinates x, y and z also correspond to the tangential, radial, and longitudinal directions in the wood.

According to Figure 5, after 120 s, the temperature of all points in the composite cell reached approximately 338.15 K over time. Moreover, the heat transfer rate was higher closer to the heating surface. Figure 6 shows the temperature distribution of composite cells 0.02 mm from the heating surface after 7.2 × 10^−^^4^, 1.2 × 10^−^^3^, 2.3 × 10^−^^3^, 9.6 × 10^−^^3^, and 1.8 × 10^−^^2^ s. The heat transfer rate of the alloy was faster than that of the wood cell wall at the beginning of heating.

**Figure 7:**
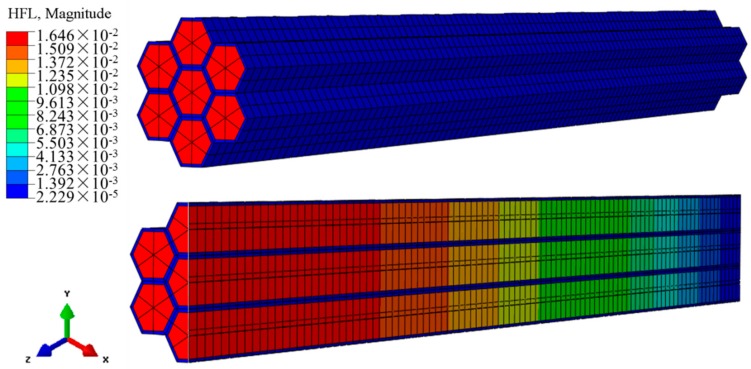
Heat flux in the model.

Figure 7 shows the heat flux for a heat transfer time at 120 s, where the heat flow through the alloy of the unit cross-sectional area was much higher than that of the wood cell wall over the same time. Hence, the heat transfer rate of the alloy was higher than that of the wood cell wall; this verifies the accuracy of the model.

### 3.3. Experimental Verification

**Figure 8:**
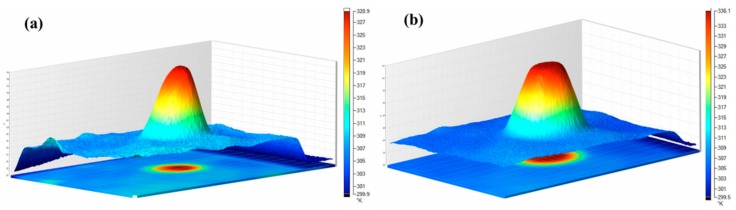
Temperature distribution of the sample. (**a**) the sample heated for 21 s and (**b**) 105 s.

The sample of φ10 × 2 mm^2^ was heated at 343.15 K using a DB-2A plate heater (Ningbo Hi-Tech Zone, Ningbo, China) and analyzed using Fluke Ti100 infrared thermography (Everett, MA, USA). Figure 8 shows that the temperature distribution of the sample reached 328.95 K and 336.15 K when heated for 21 s and 105 s, respectively.

**Figure 9:**
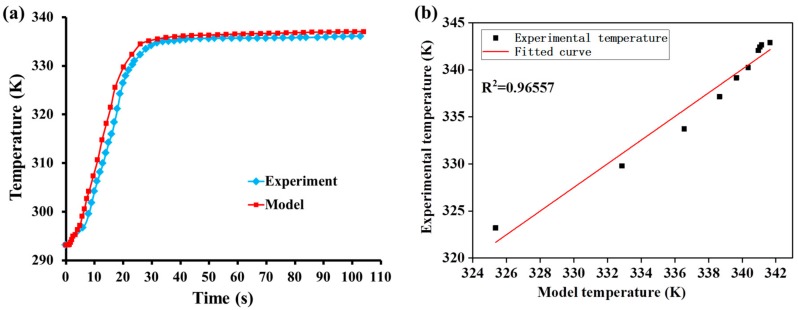
Comparison of temperature results obtained from the experiments and the model: (**a**) change of experimental and model temperature with time and (**b**) best-fit line for the results.

Figure 9a shows a comparison between the experimental values and model values; both reached the maximum temperature after approximately 42 s under the same heat conditions, and the trend of heat transfer was predominantly consistent. In Figure 9b, a correlation analysis was performed on the two temperature results, resulting in a correlation coefficient of 0.96557, which confirms the feasibility of the model. The reason for the temperature difference can be summarized as follows:

(1) When setting the cell wall thermal conductivity, we assume that the cell wall is composed entirely of cellulose. Although the cellulose content is highest in the wood cell wall, the cell wall is not completely cellulose. Moreover, the thermal conductivity of cellulose is higher than that of hemicellulose and lignin. Therefore, at the level of the wood cell wall, the temperature increases faster in the model than in the experiments.
(2) The model assumes that each cell lumen is filled with metal alloy; however, this is not necessarily true in the experiment. Therefore, at the level of the wood-metal functional composites, the temperature increases faster in the model than in the experiments.

Alloy impregnation could have changed the material properties of the single wood cell phase. Thus, the thermal conductivity of the cell wall may differ before and after impregnation.

## 4. Computational Experiments

### 4.1. Influence of Cell Geometry on Thermal Properties

**Figure 10:**
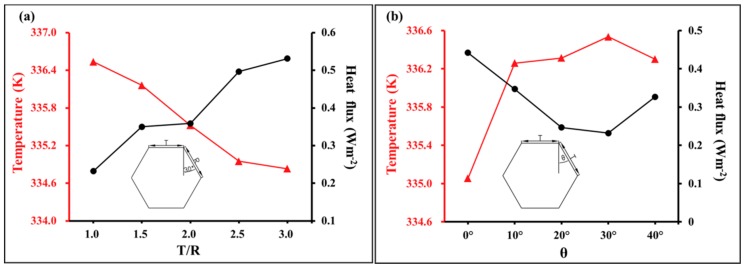
Influence of cell geometry on temperature and heat flux: (**a**) influence of the T/R ratio and (**b**) influence of angle *θ* (T/R and *θ* are defined in Figure 2).

In this section, the influence of the T/R ratio and *θ* of the cell cross-section on the temperature and heat flux of the composites is discussed, where T/R and *θ* are defined in Figure 2. *θ* was fixed at 30° to analyze the T/R ratio and T and R were fixed at 30 μm to analyze *θ*. The applied temperature was 343.15 K. Figure 10 shows the average temperature and heat flux of the last cross-section (rightmost section in Figure 4) in the longitudinal direction of the model for a heat transfer time of 60 s. Increasing the T/R ratio from 1.0 and 3.0 resulted in a decrease of the average cross-section temperature from 336.53 K to 334.83 K at 60 s (a decrease of 0.5%) and an increase of the heat flux from 0.23 W·m^−2^ to 0.53 W·m^−2^ (an increase of 130.4%). The minimum temperature difference was 0.1 K when T/R was increased from 2.5 to 3.0, and the maximum temperature difference was 0.6 K when T/R was increased from 1.5 to 2.0. The minimum heat flux difference was 0.009 W·m^−2^ when T/R was increased from 1.5 to 2.0, and the maximum heat flux difference was 0.138 W·m^−2^ when T/R was increased from 2.0 to 2.5.

Increasing *θ* from 0 to 40° first resulted in an increase then a decrease in the average temperature of the last cross-section. An increase from 335.05–336.30 K at 60 s corresponded to a change of 0.37%, which was less than the effect of increasing T/R. The heat flux first decreased then increased; a decrease from 0.44–0.33 W·m^−2^ corresponded to a change of 25.0%, which was less than that caused by increasing T/R. The maximum temperature difference was 1.2 K when *θ* was increased from 0 to 10°, and the minimum temperature difference was 0.05 K when *θ* was increased from 10° to 20°. The maximum heat flux difference was 0.1 W·m^−2^ when *θ* was increased from 10° to 20°, and the minimum heat flux difference was 0.01 W·m^−2^ when *θ* was increased from 20° to 30°.

**Figure 11:**
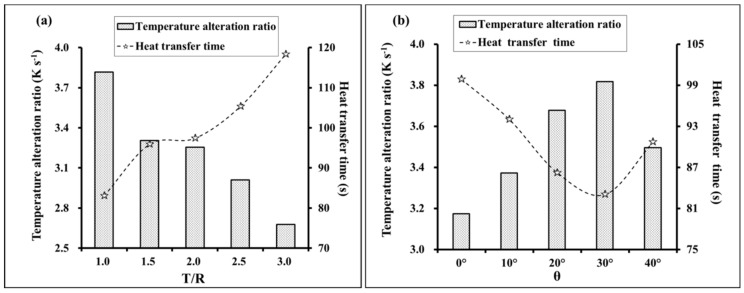
Influence of the cell geometries on the TAR (temperature alteration ratio) and heat transfer time, (**a**) influence of the ratio T/R; (**b**) influence of the angle *θ.*

Figure 11 shows the temperature alteration ratio (TAR) and heat transfer time of the last cross-section in the longitudinal direction at 343.15 K. The TAR is defined as follows, where $t_{0}$ is the initial temperature at time $\tau_{0}$ and $t_{1}$ is the temperature at $\tau_{1}$.

(14) $$TAR=\frac{t_{1}-t_{0}}{\tau_{1}-\tau_{0}}$$

For an increase in T/R from 1.0 to 3.0, the TAR decreased from 3.82–2.68 K·s^−1^ (a decrease of 29.8%) and the heat transfer time increased from 83.04–118.32 s (an increase of 42.5%). For an increase of *θ* from 0 to 40°, TAR first increased then decreased, where the increase of 3.17–3.50 K·s^−1^ represented a change of 10.4%. Conversely, the heat transfer time first decreased then increased, exhibiting a decrease of 9.2% from 99.89–90.72 s.

To clarify the relationship between thermal properties and the geometry of the cross-section cell, the area (*S*) of the unit cells with different *R*, *T*, and *θ* values was calculated, as shown in Table 2. We then investigated the effect of cell area on the thermal properties. 

**Figure 12:**
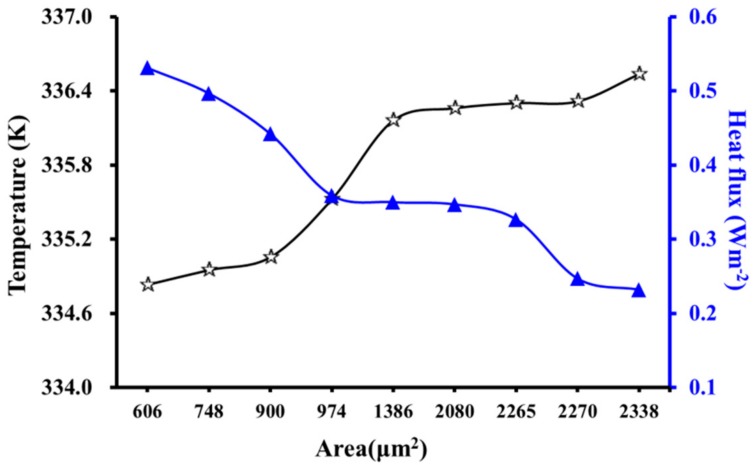
Influence of cross-sectional area on temperature and heat flux.

The observed temperature changes for different cell geometries indicated that the cross-sectional area influences the thermal behavior. Figure 12 shows that the temperature increased with increasing area of the unit cell. A similar conclusion was drawn by Lin et al. [35], who derived an equation for the temperature field distribution during the heat transfer process in anisotropic wood, where the temperature (*t*) at any point (*r*) in the wood during heat transfer could be calculated as follows:

(15) t=t1+2(t0−t1)∑n=1∞J0(μnr/R)μnJ1(μn)exp[−(λrλtλα)13·μn2τ/R2ρc]

Here, *t_0_* is the initial temperature (K); *t_1_* is the applied temperature (K); *J_0_* is the zero-order Bessel function; *J_1_* is the first-order Bessel function; and *µ_n_* is the *nth* root of the zero-order Bessel function. These values (*J_0_*, *J_1_*, and *µ_n_*) can be found in relevant mathematical manuals. In addition, *λ_r_*, *λ_t_*, and *λ_ɑ_* are the radial, tangential, and longitudinal thermal conductivities, respectively (W·m^−1^·K^−1^); *R* is the radius of the cross-section (μm); *ρ* is the density (kg·m^−3^); *c* is the specific heat (J·Kg^−1^·K^−1^); *t* is the temperature (K); and *τ* is time (s). Increasing *R,* which in this case represents the cross-sectional area of the cell, results in an increase in *t*, which is consistent with the results of our model, thereby verifying the accuracy of the model.

In addition, the cross-sectional area of the cell affects the heat flux *q* (W·m^−2^), which is defined as follows, where Q is the heat (J) and *S* is the area (μm^2^):

(16) $$q=\frac{Q}{S\times \tau }$$

Figure 12 also shows that *q* decreased with increasing *S*, which is consistent with Equation (16) where *q* is inversely proportional to *S*. This further verifies the accuracy of the model. 

**Figure 13:**
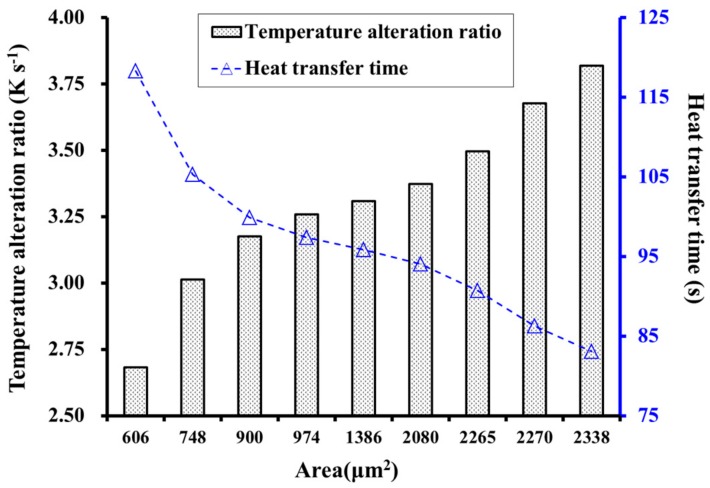
Influence of cell area on TAR and heat transfer time.

Finally, *S* has an influence on TAR and heat transfer time, where the heat flow (ϕ) is described as follows:

(17) $$\phi =\frac{\lambda S\Delta t}{\Delta X}$$

where *Δt* is the temperature difference and ΔX is the thickness. As *ϕ* is proportional to *S*, a larger heated area reduces the heat transfer time. In our model, *λ*, *Δt*, and *ΔX* are the same in all cases. The results of the model in Figure 13 show that TAR increased with increasing *S*, whereas the heat transfer time decreased. This conclusion is consistent with the description of the heat flow equation, which further verifies the accuracy of the model.

### 4.2. Influence of Heat Transfer Direction on Thermal Properties

**Figure 14:**
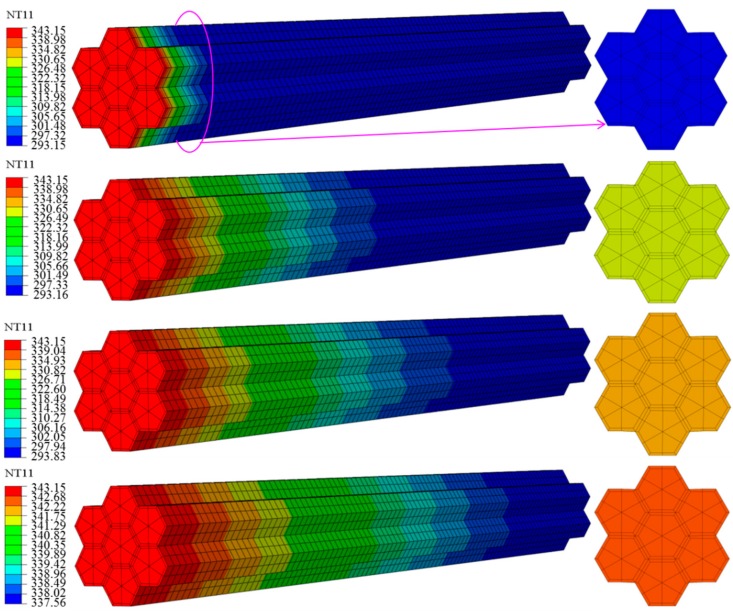
Temperature field distributions for longitudinal heat transfer.

In this section, the influence of the heat transfer direction on the temperature properties is discussed. Here, *θ* was fixed at 30°, *T* and *R* were fixed at 30 μm, and the applied temperature was 343.15 K. Figure 14 shows the temperature field distribution of a tracheid in the longitudinal direction at times of 1.2 × 10^−^^2^, 7.8 × 10^−^^1^, 1.8, and 120 s, where the 2D cross-sections on the right show the results at 0.18 mm from the heating surface. The temperature field distribution over each cross-section was consistent along the entire longitudinal direction of the tracheid. The initial temperature of 343.15 K only appeared on the first heating surface; the temperature steadily decreased with increasing distance from the initial surface. Therefore, attenuation of heat in the tracheid increased with increasing distance from the heating surface. As the heat was transferred over time, all cross-sections over the entire tracheid eventually reached 338.15 K, as shown by the 2D cross-sections, where the temperature of 293.15 K at 1.2 × 10^−^^2^ s increased to 338.15 K after 120 s.

**Figure 15:**
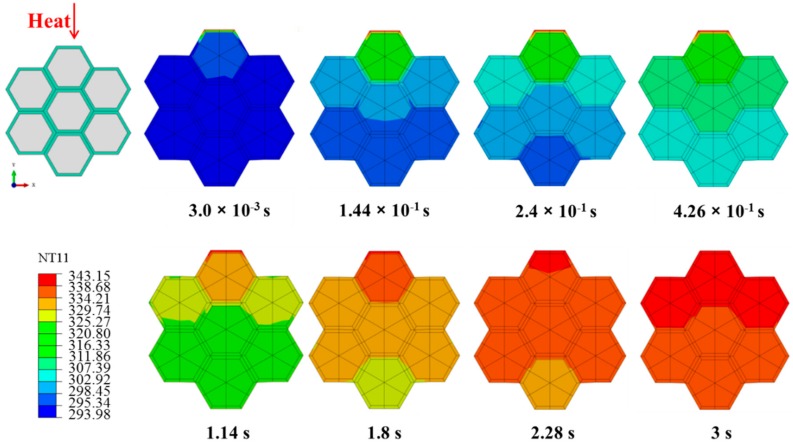
Temperature field distributions for transverse heat transfer.

Figure 15 shows the temperature field distribution of cell cross-sections (perpendicular to the longitudinal direction) after transverse heating for 3.0 × 10^−^^3^, 1.44 × 10^−^^1^, 2.4 × 10^−^^1^, 4.26 × 10^−^^1^, 1.14, 1.8, 2.28, and 3 s. The heat traveled from top to bottom along the upper surface of the tracheid, where the applied temperature of 343.15 K only appeared on the upper surface at the beginning of heating. Further from the upper surface, lower average temperatures were observed due to greater heat attenuation in the tracheid. Over time, the temperature of the lower surface increased from 293.15 K to approximately 338.15 K; eventually, the entire tracheid reached approximately 338.15 K.

**Figure 16:**
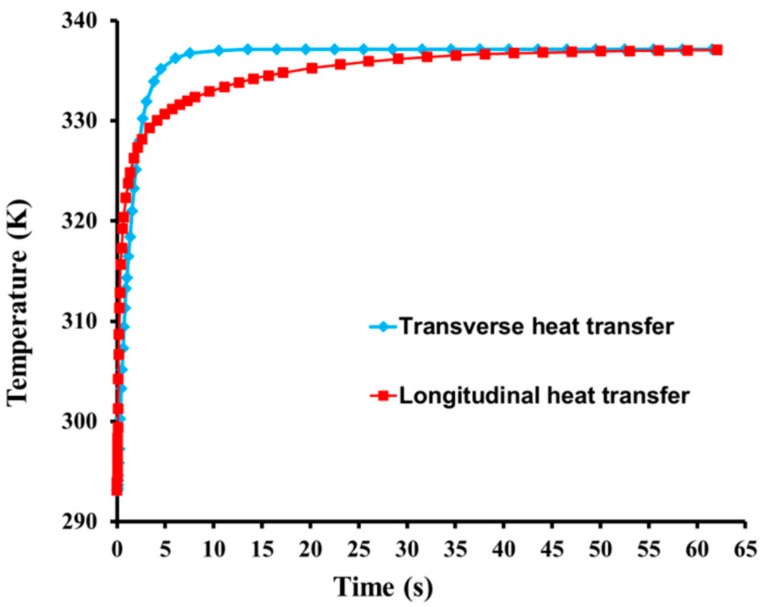
Temperature as a function of time during transverse and longitudinal heat transfer.

Figure 16 shows the temperature of the tracheid during transverse and longitudinal heat transfer as a function of time. The alloy surface close to the cell wall at the bottom was analyzed for transverse heat transfer, while the longitudinal heat transfer surface was selected from initial heating surface at 176.66 μm (equal to the transverse surface). Initially, the transverse and longitudinal heat transfer rates were the same. However, over time, the transverse heat transfer rate became higher than the longitudinal heat transfer rate; transverse and longitudinal heat transfer reached approximately 338.15 K after 7.8 s and 45.6 s, respectively. The heating area for longitudinal heat transfer was 1.64 × 10^4^ μm^2^, which was much smaller than that of transverse heat transfer (6 × 10^4^ μm^2^). As *λ*, *ΔT*, and *ΔX* were the same in both cases, Equation (17) indicates that higher heat flow is achieved for larger *S* values, allowing the material to reach the same temperature in a shorter time during transverse heat transfer than during longitudinal heat transfer.

### 4.3. Influence of Applied Temperature on Thermal Properties

**Figure 17:**
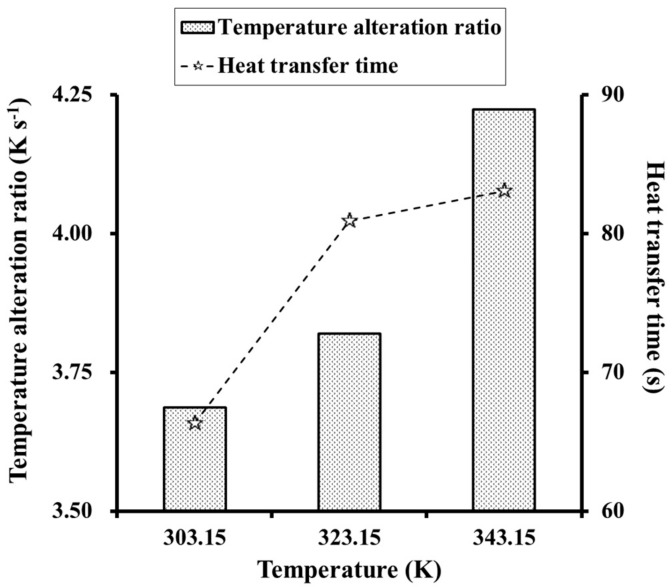
Influence of applied temperature on TAR and heat transfer time.

The influence of applied temperature on TAR and heat transfer time was studied for *θ* = 30° and *T* = *R =* 30 μm. Figure 17 shows the average temperature of the last cross-section for longitudinal heat transfer. Increasing the applied temperature from 303.15 K to 323.15 K then 343.15 K increased TAR from 3.69 K·s^−1^ to 3.82 K·s^−1^ then 4.22 K·s^−1^, respectively, corresponding to an increase of 3.5% and 10.5%, respectively. The heat transfer time also showed an increasing trend, from 66.33 s to 80.89 s then 83.04 s, corresponding to changes of 21.95% and 2.66%, respectively. Greater changes in TAR and heat transfer time were observed at lower applied temperatures. As *λ* and the cell geometry were fixed in this analysis, lower initial applied temperatures resulted in shorter equilibration times.

## 5. Conclusions

A 3D microscale heat transfer analytical model was developed for wood-metal functional composites and verified by experiments. A fitting curve was obtained for the experimental and model temperature results, which had a correlation coefficient of 0.96557, indicating the accuracy of the model. Abaqus FEM was used to analyze the temperature, heat flux, TAR, and heat transfer time of composites with various cell geometries, heat transfer directions, and applied temperatures. Increasing the cross-sectional cell area increased the temperature and TAR, but decreased the heat flux and heat transfer time. The transverse heat transfer rate was higher than the longitudinal heat transfer rate due to the effect of cell area. Lower applied temperatures resulted in lower TAR values and heat transfer times. The model was verified by several heat transfer relationships. In conclusion, the proposed method can easily and reliably simulate the thermal properties of wood materials, providing innovative opportunities for research and development.

## Figures and Tables

**Table 1:** Thermal parameters of wood cell walls and the metal alloy.

	Density (kg·m^−3^)	Thermal Conductivity (W·m^−1^·K^−1^)		Specific Heat (J·Kg^−1^·K^−1^)
Wood cell wall	1460 [30]	1 (λ_||_) [12]	0.26 ($\lambda_{⊥}$) [12]	1600 [32]
Alloy	7100	10		210

**Table 2:** Area of unit cells with different *R, T,* and *θ* values.

*θ* (°)	T (μm)	R (μm)	S (μm^2^)
30		10	606
		12	748
	30	15	974
		20	1386
		30	2338
0	T = R = 30 μm		900
10			2080
20			2270
30			2338
40			2265
